# Brain Activity Associated With Expected Task Difficulty

**DOI:** 10.3389/fnhum.2019.00286

**Published:** 2019-08-28

**Authors:** Miek J. de Dreu, Irena T. Schouwenaars, Geert-Jan M. Rutten, Nick F. Ramsey, Johan M. Jansma

**Affiliations:** ^1^Department of Neurosurgery, Elisabeth-TweeSteden Hospital, Tilburg, Netherlands; ^2^Department of Neurology and Neurosurgery, University Medical Center Utrecht Brain Center, Utrecht University, Utrecht, Netherlands

**Keywords:** fMRI, cognition, alertness, default mode network, mental preparation, brain activity, task difficulty, cue

## Abstract

Previous research shows that people can use a cue to mentally prepare for a cognitive challenge. The response to a cue has been defined as phasic alertness which is reflected in faster responses and increased activity in frontal, parietal, thalamic, and visual brain regions. We examine if and how phasic alertness can be tuned to the expected difficulty of an upcoming challenge. If people in general are able to tune their level of alertness, then an inability to tune may be linked to disease. Twenty-two healthy volunteers performed a cued visual perception task with two levels of task difficulty. Performance and brain activity were compared between these two levels. Performance was lower for difficult stimuli than for easy stimuli. For both cue types, participants showed activation in a network associated with central executive function and deactivation in regions of the default mode network (DMN) and visual cortex. Deactivation was significantly stronger for cues signaling difficult stimuli than for cues signaling easy stimuli. This effect was most prominent in medial prefrontal gyrus, visual, and temporal cortices. Activation did not differ between the cues. Our study shows that phasic alertness is represented by activated as well as deactivated brain regions. However only deactivated brain regions tuned their level of activity to the expected task difficulty. These results suggest that people, in general, are able to tune their level of alertness to an upcoming task. Cognition may be facilitated by a brain-state coupled to expectations about an upcoming cognitive challenge. Unique identifier = 842003004^1^.

## Introduction

Previous research shows that people can use a cue to mentally prepare for a cognitive challenge. If participants are instructed to mentally prepare at the moment a cue is presented, this has been defined as phasic alertness [e.g., the rapid mobilization of resources to process an expected stimulus (Nebes and Brady, 1993)]. The ability to influence our level of alertness could be important, specifically if alertness also comes with a cost. If the demand on alertness is too high, this could potentially lead to fatigue (Härmä et al., 2008) or stress-related problems. In this study, we aim to examine if and how healthy adults tune their level of alertness to the expected difficulty of an upcoming cognitive task.

If a cue is presented just before a task stimulus, people are generally able to respond faster in simple motor response tasks (Fan et al., 2002; Macleod et al., 2010; Weinbach and Henik, 2011) and perceive degraded visual stimuli more accurately (Kusnir et al., 2011). Imaging studies have shown that phasic alertness is associated with increased activation in frontal, parietal, thalamic (Shulman et al., 1999; Fan et al., 2005; Yanaka et al., 2010), temporo-occipital (Thiel et al., 2004), and visual brain regions (Bartolucci and Smith, 2011). These imaging studies have provided valuable information about the representation of phasic alertness in the brain. However, phasic alertness has generally been studied as an on/off phenomenon or analysis has been restricted to visual cortex. Therefore, it is unclear if alertness also reflects the expected difficulty of the cognitive challenge in whole brain networks.

Furthermore, previous studies have indicated that deactivation of brain regions which are part of the default mode network (DMN) may facilitate cognitive task execution. First, studies of task execution show that an increasing level of task difficulty is associated with increasing deactivation (McKiernan et al., 2003, 2006; Jansma et al., 2007; Singh and Fawcett, 2008; Pyka et al., 2009; Hedden et al., 2012; Čeko et al., 2015). Second, Jansma et al. (2007) have shown that the medial prefrontal part of the DMN only tunes to the task difficulty, which the participant could anticipate, but was not affected by the actual difficulty of each stimulus, which the participant could not anticipate. Finally, Weissman et al. (2006) found that deactivation in the DMN just before the stimulus was weaker if subjects responded relatively slow. Perhaps these subjects responded more slowly because they were temporarily less alert.

For our study, we designed a cued visual perception task with an easy and difficult condition. The cues provided information about the difficulty of the upcoming stimulus but did not provide any information that would facilitate the task itself. In half of the trials, the cue was not followed by a stimulus. Only these trials were analyzed for brain activity so we could completely isolate activation associated with the cue, from activation associated with task execution. We hypothesize that participants tune their activity to the expected difficulty of the task.

## Materials and Methods

### Participants

Participants were recruited *via* online advertisement. Participants were excluded if they reported a history of significant neurological or psychiatric disorders, or contra-indications for the magnetic resonance imaging (MRI) scan (metal objects in or around the body, claustrophobia, or pregnancy). This study was carried out in accordance with the recommendations of the Medical Research Involving Human Subjects Act (WMO), Medical Research Ethics Committee Brabant. The protocol was approved by the Medical Research Ethics Committee (protocol number: NL51147.028.14). All subjects gave written informed consent in accordance with the Declaration of Helsinki.

Twenty-two healthy right-handed volunteers participated in the study. Data from two participants were excluded due to scanner artifacts. Results are reported for the remaining 20 participants [M/F: 4/16; age ± standard error of the mean (SEM): 36 years ± 2.5, range: 19–58 years].

### Task Design

We designed a task (with event-related design) that allowed us to examine anticipation effects related to the expected difficulty of a task, without confounding effects of execution of the task.

**Figure 1:**
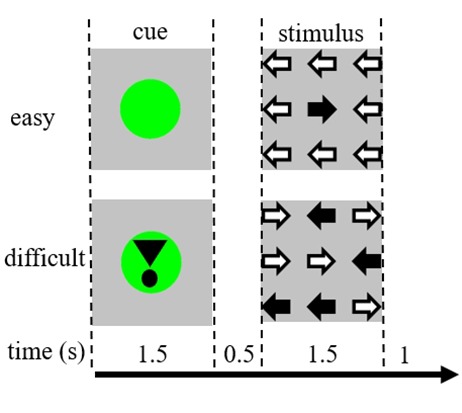
Representation of the visual stimuli. Each trial was 4.5 s. In half of the trials the cue was not followed by a stimulus. These trials were used for the functional magnetic resonance imaging (fMRI) analysis.

Task stimuli consisted of nine arrows in a three by three layout on a black screen (Figure 1). An “easy stimulus” contained eight arrows in the correct direction and one arrow in the opposite direction. A “difficult stimulus” contained five arrows in the correct direction and four arrows in the opposite direction (Figure 1). Participants were instructed to press a button with the hand corresponding to the direction of the majority of the arrows. Easy and difficult stimuli were presented in a random order. Baseline stimuli consisted of a stationary black screen with the text: “you have a 30 s break” in Dutch and in white letters. Stimuli were presented by “presentation” software.

Each trial started with a cue indicating the difficulty of the subsequently presented stimulus. A green dot indicated an “easy stimulus,” a green dot with an exclamation mark indicated a “difficult stimulus” (Figure 1).

In half of the trials, the cue was not followed by a stimulus, in the other half the stimulus was presented 500 ms after the cue disappeared. This resulted in four conditions: (1) a cue for an easy stimulus, not followed by a stimulus (“CueE”); (2) a cue for a difficult stimulus, not followed by a stimulus (“CueD”); (3) a cue for an easy stimulus, followed by an easy stimulus (“StimE”); and (4) a cue for a difficult stimulus, followed by a difficult stimulus (“StimD”). All conditions were contrasted to baseline. Functional MRI (fMRI) results were based on CueE and CueD. Performance results were based on StimE and StimD. CueE and CueD were presented in a pseudo-randomized order using M sequences to maximize general linear model (GLM) regressor independence between conditions and optimize design efficiency (Buracas and Boynton, 2002). The participants could not predict if a cue would be followed by a stimulus or not.

The duration of each trial was 4,500 ms. Cues were presented at *t* = 0 for 1,500 ms and stimuli were presented at *t* = 2,000, also for 1,500 ms (Figure 1). The task was performed in two runs of 68 trials. Baseline consisted of three 30 s rest periods, before, after, and between the two runs. The total duration of the task was 11.7 min. This was the second task of the session and started after the participants were lying approximately 10 min in the scanner.

Participants were instructed to prepare for an easy or difficult stimulus based on the information of the cue (Figure 1) and to indicate as accurately and quickly as possible the direction of the majority of the arrows in the stimulus. Participants practiced the task outside the scanner following a standard practice protocol of 3 min. This protocol was repeated if performance was insufficient (below 70% accuracy for the difficult stimuli).

A mirror attached to the head coil enabled participants to see a see-through projection screen positioned behind the head. A video projector inside the scanner room projected the task stimuli on the screen. Two pneumatic push-button boxes with air pressure were used to record responses. Participants used the left thumb or index finger for answering “left” and the right for answering “right.”

### Image Acquisition

Scans were performed on a 3T Philips Achieva scanner (Philips Medical Systems, Best, Netherlands) using a 32-channel SENSE head coil. A 3D T1-weighted structural image was acquired for anatomical registration purposes [scan parameters: TR/TE: 8.4/3.8 ms, FOV: 254 × 254 × 158 mm^3^, flip angle: 8°, voxel size 1 mm isotropic, whole brain coverage, 158 slices (sagittal orientation)]. fMRI images were obtained using a 3D PRESTO pulse sequence [Liu et al., 1993; van Gelderen et al., 1995; Neggers et al., 2008; scan parameters: volume acquisition time 1.5 s, TR/TE: 19/27 ms, FOV: 256 × 256 × 160 mm^3^, flip angle: 10°, voxel size 4 mm isotropic, whole brain coverage, 40 slices (sagittal orientation), 370 volumes]. Six dummy scans were acquired and then discarded by the scanner.

### Image Pre-processing

fMRI data were preprocessed using statistical parametric mapping (SPM12; Wellcome Trust Centre for Neuroimaging, University College London, London, UK^2^). Scans from one session were realigned to the first scan to correct for subject movement using a least squares approach, a six parameter (rigid body) spatial transformation, and a 2nd degree B-spline estimation. The scans were co-registered to the T1 using a rigid body model. The parameters were estimated by the normalized mutual information function. The images were resliced by a 4th degree B-spline. The T1 was spatially normalized into standard MNI-space using very light bias regularization (0.0001) and a 4th degree B-Spline. The resulting parameters were applied to all functional scans in order to minimize anatomical differences and therefore enable group analysis. Finally, all scans were spatially smoothed with a 3D Gaussian filter (full-width at half-maximum: 8 mm) to further minimize the effect of functional anatomical differences.

### Individual fMRI Analysis

Event-related GLM regression analysis was performed for each voxel to generate individual activation maps using a mass-univariate approach with a global approximate AR(1) autocorrelation model, and a high pass filter (128 s cut-off). Baseline was not explicitly modeled. The basic function was a canonical HRF without derivatives. A masking threshold of 0.8 was used. Separate regressors were used for each condition (CueE, 17 timepoints; CueD, 16 timepoints; StimE, 17 timepoints; StimD, 18 timepoints; and a nuisance regressor for blanc periods in the task related to the m-sequence, 68 timepoints). No timepoints were excluded from the analysis. Beta maps were transformed to reflect the actual percentage signal change in each voxel. We only present the results for the CueE and CueD regressors, reflecting signal changes for cues without a stimulus. Beta and statistical *t*-maps were checked visually for major artifacts.

### Group fMRI Analysis

A second level fMRI analysis was performed with linear regression at each voxel for visualization and region of interest (ROI) selection. A ROI analysis was performed with GNU data language (GDL^3^), using individual subject percentage signal change maps generated by the GLM analysis. The voxels within a ROI or network were averaged to calculate the signal difference between CueE, CueD, and baseline.

### ROI Selection

Supratentorial local maxima and minima were determined for both CueE and CueD by SPM with an uncorrected threshold (*p* < 0.001; Table 1). Cubic ROIs of predefined size and shape were placed over the local maxima for the CueE–baseline and CueD–baseline contrast separately within a predefined raster (Jansma and Rutten, 2017; Table 2). ROIs were included in the analysis if they contained a significant local maximum for CueE or CueD. By defining ROIs on local maxima for both conditions separately we prevent bias towards either CueE or CueD. Furthermore, the predefined shape and size of the ROIs within a raster reduces the effect of circularity, because the borders of these ROIs are not affected by noise (Kriegeskorte et al., 2009). Furthermore, this method facilitates quantitative comparison of results between conditions, and potentially between different studies, thus facilitating quantitative reproducibility of fMRI results. Placement of the ROIs over the local maxima optimized power. However, the location of the activity peak is known to be affected by noise, therefore the exact location of the ROIs may always reproduce for each ROI. This does not affect the network results since all ROIs are averaged and exact location, therefore, is not as relevant.

**Table 1:** Activity pattern.

TIA	(2,305 voxels)		MNI coordinates of peak activity					
			CueE			CueD		
ROI no	ROI name	Abb	*x*	*y*	*z*	*x*	*y*	*z*
1	Left inferior frontal gyrus, opercular part	LIFGop	−40	8	24	−40	8	32
2	Left precentral gyrus	LPCG	−40	0	52			
3	Left inferior parietal gyrus	LIPG	−36	−48	44	−36	−44	40
4	Left middle frontal gyrus	LMFG				−32	56	20
5	Left inferior frontal gyrus, triangular part	LIFGtri	−32	28	0	−32	28	−4
6	Left middle frontal gyrus	LMFG				−44	40	12
7	Left superior temporal gyrus	LSTG	−64	−48	8			
8	Left middle frontal gyrus	LMFG	−44	20	44			
9	Left supramarginal gyrus	LSMG	−52	−44	28			
10	Right inferior frontal gyrus, opercular part	RIFGop	40	8	28	44	16	28
11	Right precuneus	RPCUN	28	−60	36			
12	Right inferior parietal gyrus	RIPG				32	−52	44
13	Right superior frontal gyrus, medial part	RSFGmp				8	44	44
14	Right middle temporal gyrus	RMTG	60	−44	8	64	−44	8
15	Right inferior temporal gyrus	RITG				52	−24	−12
16	Right basal ganglia, thalamic part^1^	RBGtp	4	−20	32			
17	Right hippocampus	RHIP	28	−20	−20			
18	Right insula^1^	RINS^1^	48	20	4			
19	Right hippocampus	RHIP	40	−12	−16			
20	Right inferior temporal gyrus	RIFG	56	−52	−16			
**TID**	**(1,284 voxels)**
21	Left medial prefrontal gyrus^1^	LMPFG				−8	64	8
22	Left superior parietal gyrus	LSPG				−20	−44	72
23	Left calcarine fissure	LCALC	−8	−64	12			
24	Left calcarine fissure	LCALC	−8	−84	−4			
25	Left angular gyrus^1^	LAG^1^				−36	−80	28
26	Left superior temporal gyrus	LSTG				−56	−28	20
27	Left rolandic operculum	LROLop				−56	0	12
28	Right cuneus	RCUN	4	−84	24/36	8	−84	36
29	Right calcarine fissure	RCALC	12	−84	0	12	−84	0
30	Right angular gyrus^1^	RAG^1^	48	−72	32			
31	Right angular gyrus	RAG				44	−76	32

**Figure 2:**
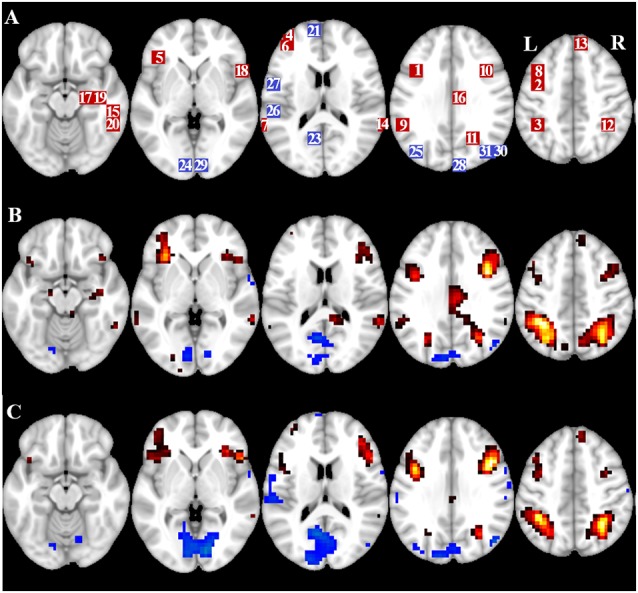
Overview of region of interest (ROI) location and whole brain fMRI results. **(A)** ROIs for task induced activation (red) and task induced deactivation (blue) note: ROI 22 is not displayed because the location is outside the chosen slices. MNI coordinates, BA number, and size of the ROIs can be found in Table 2. **(B)** Visual representation of the *t*-values for CueE vs. baseline (≥3 = red to yellow, ≤ −3 = blue to light blue), **(C)** *T*-values for CueD vs. baseline (≥3 = red to yellow, ≤ −3 = blue to light blue). Images are in neurological orientation (L = left, R = right), names, abbreviations, MNI coordinates of the peak activations voxel can be found in Table 1.

*MNI coordinates of the peak activated voxel used to select each ROI. Some ROIs overlap with the same AAL region, therefore received the same name and abbreviation (e.g., ROI 4, 6, and 8). The numbers as well as Figure 2 can be used to distinguish these ROIs. Abbreviations: TIA, task induced activation network; MNI, Montreal Neurological Institute; CueE, a cue indicating an easy stimulus, not followed by a stimulus; CueD, a cue indicating a difficult stimulus, not followed by a stimulus; ROI, region of interest; abb, abbreviation; TID, task induced deactivation network, ^1^labeled by approximation because AAL classification was not sufficient*.

**Table 2:** Region of interest (ROI) characteristics.

TIA (2,305 voxels)				MNI coordinates		
no	Abb	BA	NV	*x*	*y*	*z*
1	LIFGop	44	125	−39	15	30
2	LPCG	6	125	−39	0	45
3	LIPG	40	125	−39	−45	45
4	LMFG	46	47	−39	60	15
5	LIFGtri	47	125	−39	30	0
6	LMFG	45	122	−39	45	15
7	LSTG	22	52	−69	−45	15
8	LMFG	9	124	−39	15	45
9	LSMG	48	125	−54	−45	30
10	RIFGop	48	125	39	15	30
11	RPCUN	0	125	24	−60	30
12	RIPG	40	125	39	−45	45
13	RSFGmp	9	120	9	45	45
14	RMTG	22	94	69	−45	15
15	RITG	20	123	54	−30	−15
16	RBGtp	na	125	9	−15	30
17	RHIP	20	125	24	−15	−15
18	RINS	48	123	54	15	0
19	RHIP	20	125	39	−15	−15
20	RIFG	20	125	54	−45	−15
**TID (1,284 voxels)**						
21	LMPFG	10	125	−9	60	15
22	LSPG	na	102	−24	−45	75
23	LCALC	17	125	−9	−60	15
24	LCALC	17	125	−9	−90	0
25	LAG	39	124	−39	−75	30
26	LSTG	42	125	−54	−30	15
27	LROLop	48	125	−54	0	15
28	RCUN	na	119	9	−90	30
29	RCAL	17	125	9	−90	0
30	RAG	na	66	54	−75	30
31	RAGlp	19	123	39	−75	30

*MNI coordinates represent the middle voxel of each ROI. Abbreviations: ROI, region of interest; TIA, task induced activation network; abb, abbreviation; BA, Brodmann area; NV, number of voxels (size); TID, task induced deactivation network; For ROI names see Table 1*.

ROIs over regions with an increase in signal change were combined in a task-induced activation network (“TIA”). ROIs over regions with a decrease in signal change were combined in a task-induced deactivation network (“TID”; Tables s 1, 2). Where possible, the ROI names were determined using the AAL atlas (Tzourio-Mazoyer et al., 2002).

Table 2 and Figure 2 provide an overview of the size and location of all ROIs. Because the size and borders of the ROIs are predefined, it is possible that the average activity in the ROI is not significantly different from baseline, for example because the number of activated voxels within the ROI is relatively small. Although the average activity at network level is significantly different from baseline for all conditions (Table 3), this is not the case for eight of the 31 individual ROIs for CueE and three of 31 ROIs for CueD (Table 4). Only voxels that contained signal for every participant were included in the analysis. The maximum size of an ROI is 125 voxels, some ROIs include less than 125 voxels because they are positioned near the skull.

**Table 3:** Network results.

	TIA				TID			
Condition	Mean	SEM	*t*-score	*p*-value	Mean	SEM	*t*-score	*p*-value
CueE	0.12	0.02	6.11	<0.001	−0.07	0.02	2.68	0.007
CueD	0.12	0.02	7.61	<0.001	−0.12	0.02	5.37	<0.001
CueD–CueE			−0.24	0.408			**−2.48**	**0.011**

*TIA and TID network results for contrasts: CueE–baseline, CueD–baseline, and CueD–CueE. Statistical results for CueD–baseline and CueE–baseline are only intended for illustrative purpose, as ROIs are selected based on these two contrasts. Abbreviations: TIA, task induced activation network; TID, task induced deactivation network; SEM, standard error of the mean; CueE, a cue indicating an easy stimulus, not followed by a stimulus; CueD, a cue indicating a difficult stimulus, not followed by a stimulus. Bold values indicate significant results for the most important contrast*.

**Table 4:** ROI results.

TIA		CueE–baseline				CueD–baseline				CueD–CueE	
no	Abb	Mean	SEM	*t*-score	*p*-value	Mean	SEM	*t*-score	*p*-value	*t*-score	*p*-value
1	LIFGop	0.14	0.04	3.90	<0.001	0.15	0.04	4.00	<0.001	−0.29	0.388
2	LPCG	0.13	0.03	4.21	<0.001	0.15	0.03	4.68	<0.001	−0.83	0.207
3	LIPG	0.22	0.03	6.67	0.000	0.17	0.03	5.00	<0.001	−1.57	0.066
4	LMFG	0.09	0.03	2.92	0.004	0.12	0.03	3.79	<0.001	−1.44	0.082
5	LIFGtri	0.14	0.03	4.52	<0.001	0.18	0.03	5.40	<0.001	−1.14	0.135
6	LMFG	0.11	0.04	2.81	0.005	0.15	0.04	3.57	<0.001	−1.02	0.161
7	LSTG	0.05	0.03	1.58	0.065	−0.01	0.03	0.26	0.400	**−2.68**	**0.007**^1^
8	LMFG	0.11	0.03	3.18	0.002	0.12	0.03	3.40	0.001	−0.35	0.363
9	LSMG	0.11	0.04	3.04	0.003	0.07	0.04	1.95	0.032	−1.07	0.149
10	RIFGop	0.21	0.04	5.80	0.000	0.23	0.04	6.05	0.000	−0.64	0.265
11	RPCUN	0.15	0.03	5.04	<0.001	0.15	0.03	4.98	<0.001	−0.18	0.431
12	RIPG	0.17	0.04	4.18	<0.001	0.17	0.04	4.08	<0.001	0.00	0.498
13	RSFGmp	0.13	0.03	3.77	<0.001	0.15	0.04	4.24	<0.001	−0.96	0.174
14	RMTG	0.09	0.02	4.94	−0.001	0.06	0.02	3.41	0.001	−1.05	0.154
15	RITG	0.11	0.03	3.60	<0.001	0.09	0.03	2.93	0.004	−0.55	0.294
16	RBGtp	0.12	0.03	3.87	<0.001	0.07	0.03	2.23	0.019	−1.25	0.113
17	RHIP	0.09	0.04	2.11	0.048	0.09	0.04	2.16	0.022	−0.11	0.457
18	RINS	0.13	0.05	2.63	0.008	0.13	0.05	2.66	0.007	−0.07	0.473
19	RHIP	0.11	0.03	3.75	<0.001	0.09	0.03	2.83	0.005	−0.68	0.253
20	RIFG	0.10	0.03	2.78	0.006	0.05	0.04	1.44	0.083	−1.33	0.099
**TID**											
21	LMPFG	−0.03	0.05	−0.64	0.266	−0.13	0.05	0.27	0.008	**−1.83**	**0.041**
22	LSPG	−0.04	0.02	−1.57	0.066	−0.07	0.02	0.07	0.004	**−2.32**	**0.016**
23	LCALC	−0.11	0.03	−3.20	0.002	−0.14	0.03	0.00	<0.001	−1.07	0.149
24	LCALC	−0.14	0.07	−2.05	0.027	−0.19	0.07	0.03	0.006	−1.62	0.061
25	LAG	−0.05	0.04	−1.28	0.108	−0.09	0.04	0.11	0.030	−0.92	0.184
26	LSTG	−0.04	0.04	−1.08	0.146	−0.15	0.04	0.15	<0.001	**−2.69**	**0.007**
27	LROLop	−0.04	0.03	−1.24	0.115	−0.09	0.03	0.11	0.004	−1.43	0.085
28	RCUN	−0.13	0.06	−2.30	0.016	−0.16	0.05	0.02	0.002	−0.98	0.170
29	RCALC	−0.09	0.08	−1.14	0.135	−0.22	0.08	0.13	0.007	**−2.90**	**0.004**
30	RAG	−0.07	0.02	−4.22	<0.001	−0.10	0.02	0.00	<0.001	−0.94	0.178
31	RAG	0.01	0.04	0.33	0.372	−0.01	0.04	0.37	0.382	−0.79	0.220

*Individual ROI reults for contrasts CueD–baseline, CueE–baseline as well as CueD–CueE. Statistical results for CueD–baseline and CueE–baseline are only intended for illustrative purpose, as ROIs are selected based on these two contrasts. Abbreviations: ROI, region of interest; abb, abbreviation; TIA, task induced activation network; TID, task induced deactivation network; for ROI names see Table 1. ^1^This significant contrast does not survive Bonferroni correction, which is set at: 0.0025*.

### Hypotheses Testing

We tested the following hypotheses in this study:

Hypothesis 1: TIA will show increased signal change for CueD, compared to CueE.

Hypothesis 2: TID will show decreased signal change for CueD, compared to CueE.

### Statistical Analysis

Power analysis was not performed since it was the first time this specific task design was presented to participants. Therefore there is no knowledge of the expected percent signal change and variability of the conditions (Desmond and Glover, 2002).

Task accuracy was calculated for StimE and StimD as the percentage of correct responses. Reaction times were calculated for StimE and StimD over all correct responses.

One sample *t*-tests were used to compare the percentage signal change for TIA and TID compared to baseline for CueE and CueD. Paired sample one-sided *t*-tests were used to compare signal change, accuracy, and reaction time differences between CueD and CueE (Hypothesis 1 and 2).

*Post hoc* testing involved separate *t*-tests for each ROI within each network. ROI-analysis was Bonferroni corrected if the network omnibus test was not significant. We present uncorrected *p*-values and a comment stating if this *p*-value would have survived Bonferroni correction for all multiple comparisons. SPSS 24 was used for all statistical analyses.

## Results

### Performance

**Figure 3:**
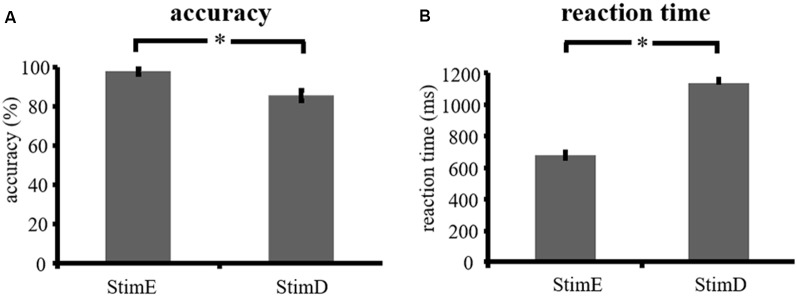
Performance results. **(A)** Accuracy for both stimulus categories, **(B)** reaction time for both stimulus categories. Error bars represent standard error of the mean (SEM). *p ≤ 0.001. Abbreviations: StimE, a cue indicating an easy stimulus, followed by an easy stimulus; StimD, a cue indicating a difficult stimulus, followed by a difficult stimulus.

The accuracy for the easy stimuli (“StimE”) was 98% ± 1.2% SEM and for difficult stimuli (“StimD”) 85% ± 2.6%. The reaction time for StimE was 682 ± 20 ms and for StimD 1,138 ± 27 ms. Both conditions show sufficient accuracy rates to be confident that the task was executed as instructed by all participants. As hypothesized, the participants responded less accurate (*T* = −4.3, *p* < 0.001) and slower (*T* = 17.8, *p* < 0.001) for StimD than for StimE (Figure 3), indicating that the contrast between the easy and difficulty stimuli was successful.

### Functional MRI

#### Descriptives

The TIA network consisted of 20 ROIs, of which 10 were based on local maxima for CueE, five were based on local maxima for CueD, and five contained both a CueE and a CueD maximum (Table 1). Of these 20 ROIs, 19 displayed significant activity compared to baseline for CueE, 18 for CueD (Table 1). The average signal change in TIA compared to baseline for CueE was 0.12 ± 0.02 (*t* = 6.11, *p* < 0.001) and for CueD 0.12 ± 0.02 (*t* = 7.61, *p* < 0.001).

The TID network consisted of 11 ROIs, of which three were based on local maxima for CueE, six were based on local maxima for CueD, and two contained both a CueE and a CueD maximum (Table 1). Of these 11 ROIs, four displayed significant activity compared to baseline for CueE, 10 for CueD (Table 1). The average signal change in TID compared to baseline for CueE was −0.07 ± 0.02 (*t* = 2.68, *p* < 0.01), and for CueD −0.12 ± 0.02 (*t* = 5.37, *p* < 0.001; Table 3).

#### Tuning to the Expected Difficulty

**Figure 4:**
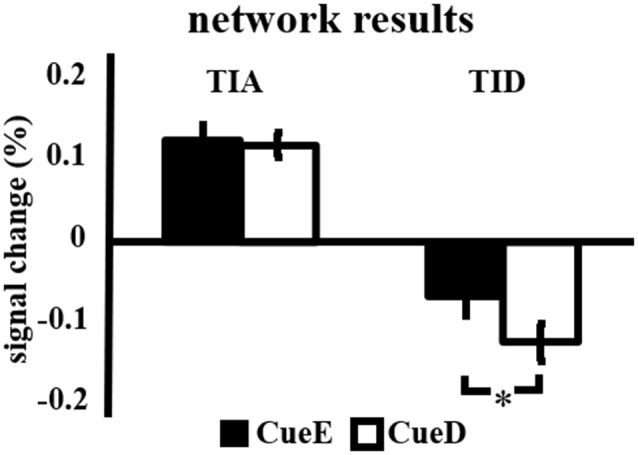
Overview of the network results. Error bars represent SEM. *p ≤ 0.05 for cue difficult vs. cue easy. Abbreviations: TIA, task induced activation network; TID, task induced deactivation network; CueE, a cue indicating an easy stimulus, not followed by a stimulus; CueD, a cue indicating a difficult stimulus, not followed by a stimulus.

**Figure 5:**
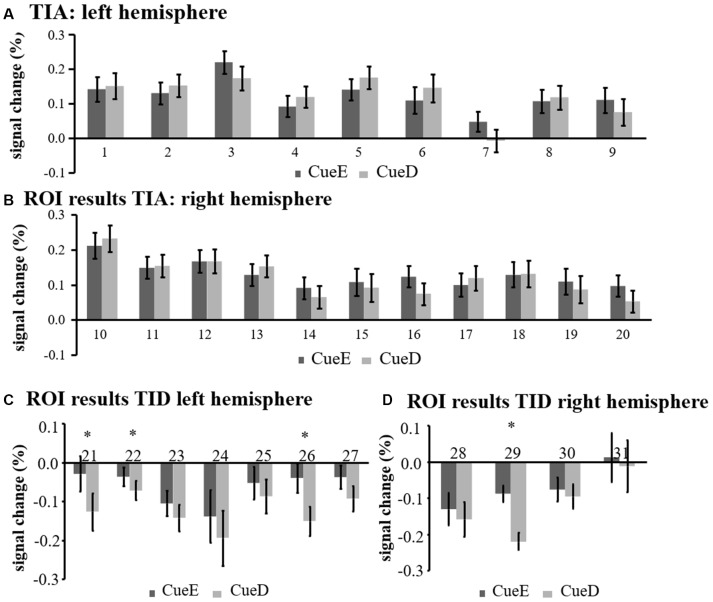
Overview of individual ROI responses within the task induced activation network, left **(A)** and right hemisphere **(B)**, and task induced deactivation, left **(C)**, and right hemisphere **(D)**. Error bars represent SEM. *p ≤ 0.05 for CueD vs. CueE, see Figure 2 for the location of the ROIs, see Table 1 for the names, abbreviations, and MNI coordinates of the ROIs.

The signal increase in TIA was not significantly stronger for CueD compared to CueE (*t* = −0.24, *p* = 0.41) indicating that the level of TIA activation is not tuned to the expected difficulty (Figure 4, Table 3). *Post hoc* ROI analysis showed none of the 20 ROIs within TIA with a significant signal increase for CueD compared to CueE after applying Bonferroni correction (Table 4, Figure 5).

The signal decrease in TID was significantly stronger for CueD compared to CueE (*T* = −2.48, *p* = 0.01) indicating that the level of TID is tuned to the expected difficulty (Table 3, Figure 4). *Post hoc* ROI analysis showed four out of 11 ROIs of TID with a significant signal decrease for CueD compared to CueE, namely LMPFG (*T* = −1.83, *p* = 0.04), LSPG (*T* = −2.32, *p* = 0.02), LSTG (*T* = −2.69, *p* < 0.01), and RCALC (*T* = −2.90, *p* < 0.01). These results indicate that tuning to the expected difficulty is strongest in left frontal, temporal, and visual ROIs (Table 4, Figure 5).

## Discussion

In this study, we examined if and how phasic alertness is tuned to the expected difficulty of an upcoming cognitive challenge. Phasic alertness was reflected in activated as well as deactivated brain regions. The activated network included regions of the central executive network. The deactivated network included regions of the DMN, as well as visual cortices. The level of deactivation scaled with the expected difficulty of the upcoming stimulus, while the level of activation did not. These results suggest that modulation of phasic alertness is predominantly reflected in graded suppression processes that are irrelevant for the current task (for example cardio-vagal, auditory, and visual processes). Possibly, this occurred because these processes could interfere with upcoming cognitive challenges.

Previous imaging studies of phasic alertness used one level of difficulty, only presented activated brain regions (Shulman et al., 1999; Sturm and Willmes, 2001; Thiel et al., 2004; Fan et al., 2005; Périn et al., 2010; Yanaka et al., 2010), or focused solely on activity in visual cortex (Bartolucci and Smith, 2011). These studies have identified a mostly right-lateralized fronto-parietal-thalamic network, sometimes combined with motor and visual cortical regions, that was activated by a cue. There are substantial differences in the activated networks between studies. Some of the differences between the networks in previous studies may be explained by the task that followed the cue. For instance, Thiel et al. (2004) only identified activation in visual cortical regions using a visual perception task, while Fan et al. (2005) and Yanaka et al. (2010) identified thalamic and motor activation with a motor response task. Although Bartolucci and Smith (2011) present an elegant cued task design with four levels of difficulty of an orientation discrimination task, they only presented activation in the visual cortex. Therefore, it is unclear how other brain regions responded in this task. The task-induced activation network, identified in the current study is most similar to the network found by Shulman et al. (1999), that used a similar type of visual processing task.

It is unclear if phasic alertness is a bottom-up or top-down response to an uninformative cue (Thiel et al., 2004; Hackley, 2009; Périn et al., 2010; Bartolucci and Smith, 2011; Chica et al., 2016). Phasic alertness has been considered by some as a bottom-up (Sturm et al., 1999; Hackley, 2009), by others as top-down (Bartolucci and Smith, 2011), and by others as a combination (Thiel et al., 2004; Périn et al., 2010) response. We argue that it is most suitable to label the brain response to a cue as a combination of bottom-up and top-down processing because participants both respond to an external cue and an instruction associated with this cue. However, our main results concern the difference in response to two similar cues indicating a different type of task stimulus. Therefore, we argue that the resulting difference between the cue indicating a difficult task and the cue indicating an easy task may represent a level of top-down control related to the interpretation of the cue and associated instruction.

The task induced deactivation network included brain regions of the DMN, namely the medial prefrontal gyrus, superior temporal gyri, and angular gyri (Shulman et al., 1999; Raichle et al., 2001; Raichle, 2015). It is commonly hypothesized that DMN deactivation aids task performance by reducing internal processes that interfere with cognitive challenges (Raichle et al., 2001; Buckner et al., 2008; Raichle, 2015). This view is supported by studies showing that the level of DMN deactivation scales with the difficulty of a task during task execution (McKiernan et al., 2003; Singh and Fawcett, 2008). These studies examined deactivation during task execution. Our results suggest that this scaling can already occur during the task anticipation phase. Weissman et al. (2006) examined the variability in individual reaction times to identify lapses of attention. The lapses of attention could be associated with reduced DMN deactivation. While (Weissman et al., 2006) looked at natural variation in the level of alertness, our results indicate that deactivation in several DMN regions can also be tuned in a top-down manner before the task stimulus is presented.

The most profound tuning effects were found in the left medial prefrontal, temporal and visual regions. Below we discuss possible implications of these effects for these regions in relation to their proposed function.

The medial prefrontal cortex activity has previously been associated with mind-wandering (Bertossi et al., 2017), memory retrieval (Euston et al., 2012), and cardiovagal control (Wong et al., 2007). This medial prefrontal deactivation suggests that any of these functions is already suppressed in anticipation of a cognitive challenge.

The tuning of deactivation to the expected difficulty in visual and temporal regions may be related to suppression of irrelevant visual and auditory sensory input. The tuning in the bilateral temporal regions may specifically be related to suppression of the noise generated by the MRI scanner. It is reasonable to expect that the level of sound is similar between the conditions. The fact that the level of deactivation is stronger for the difficult cues suggests that the processing of ambient sound is suppressed more strongly if a difficult task is expected. Deactivation in visual cortices is less commonly reported than deactivation in the temporal regions. However, Smith et al. (2000) have previously reported an association between widespread deactivation in the visual cortex and attention to a specific part of a picture. Giesbrecht et al. (2006) also reported modulation of visual activation associated with covert attention to objects near the center of their visual field compared to objects in the peripheral visual field. These findings indicate that the activation in the visual cortices can be modulated by covert attention to either foveal or peripheral regions. Our study adds a new dimension to these findings, as it suggests that activation in the visual cortices is not only modulated by the location of covert visual attention, but also by the expected difficulty of a visual challenge; and since the cues were identical in location and size this process seems regulated in a top-down fashion.

We also identified a set of brain regions that showed increased activation in anticipation of a cognitive challenge. This network showed similarity to the central executive network that has previously been associated with the execution of working memory tasks (Lawrence et al., 2003). However, activation in this network was not tuned to the expected task difficulty. This suggests that these regions represent processes that are similar for the difficult and easy condition. Possibly, activity in this network is associated with evaluation of the cue, or retrieval and maintenance of the task context and instructions.

Some limitations need to be taken into consideration that may affect the interpretation of our results. First, we did not include trials with stimuli that were not preceded by a cue, therefore it is not possible to examine the effect of the cue on cognitive performance. Due to time restriction, it was not possible to repeat our task using identical stimuli but without cues.

Second, we used an exclamation mark as part of the cue indicating a difficult cognitive challenge because this symbol is typically used if there is a need for increased alertness. It is possible that the exclamation mark itself may have played a role in the different activation patterns for the easy and difficult condition, due to its inherent meaning. This does, however, not affect the main interpretation of our results, except that our main results may be classified as a bottom-up response instead of a top-down response. Finally, our experimental design does not allow us to examine if the activity related to phasic alertness can be generalized to other domains, or if it is domain-specific, as it only included a visuo-perceptual cognitive challenge.

To conclude, in this study we have shown that the tuning of phasic alertness is represented by the level of deactivation in several regions. This effect is strongest in the medial prefrontal, visual, and temporal cortex which may reflect a suppression of cardiovagal control, visual processing in the peripheral visual field, and suppression of MRI scanner noise. These results suggest that cognitive performance is facilitated by a state of the brain that is tightly coupled to expectations about the difficulty of an upcoming cognitive challenge.

## Data Availability

Raw data were generated at the Elisabeth-Tweesteden hospital. Derived data that support the findings of this study are available from the corresponding author upon reasonable request.

## Ethics Statement

This study was carried out in accordance with the recommendations of Dutch social support act, medical Ethical committee Brabant. All subjects gave written informed consent in accordance with the Declaration of Helsinki. The protocol was approved by the medical ethical committee Brabant.

## Author Contributions

MD and JJ wrote the main manuscript and prepared the figures. MD and IS performed the experiments. G-JR, NR, and JJ jointly supervised the work. All authors reviewed the manuscript.

## Conflict of Interest Statement

The authors declare that the research was conducted in the absence of any commercial or financial relationships that could be construed as a potential conflict of interest.

## Footnotes

^2^http://www.fil.ion.ucl.ac.uk/spm/software/spm12/

^3^https://github.com/gnudatalanguage/gdl

## Abbreviations

DMN: default mode network
M: male
F: female
GLM: general linear model
SPM: statistical parametric mapping
CueE: a cue indicating an easy stimulus, not followed by a stimulus
CueD: a cue indicating a difficult stimulus, not followed by a stimulus
StimE: a cue indicating an easy stimulus, followed by an easy stimulus
StimD: a cue indicating a difficult stimulus, followed by a difficult stimulus
MRI: magnetic resonance imaging
ROI: region of interest
GDL: GNU data language
TIA: task induced activation network
TID: task induced deactivation network
SEM: standard error of the mean.
